# The role of MMP-9 in the anti-angiogenic effect of secreted protein acidic and rich in cysteine

**DOI:** 10.1038/sj.bjc.6605538

**Published:** 2010-01-19

**Authors:** P Bhoopathi, C Chetty, M Gujrati, D H Dinh, J S Rao, S S Lakka

**Affiliations:** 1 Program of Cancer Biology, Department of Cancer Biology and Pharmacology, University of Illinois College of Medicine at Peoria, One Illini Drive, Peoria, IL 61605, USA; 2 Department of Pathology, University of Illinois College of Medicine at Peoria, One Illini Drive, Peoria, IL 61605, USA; 3 Department of Neurosurgery, University of Illinois College of Medicine at Peoria, One Illini Drive, Peoria, IL 61605, USA

**Keywords:** angiogenesis, SPARC, VEGF, MMP-9, CD-31

## Abstract

**Background:**

Secreted protein acidic and rich in cysteine (SPARC), a matricellular glycoprotein, modulates cellular interaction with the extracellular matrix and is capable of altering the growth of various cancers. We therefore sought to determine the effect of SPARC expression on medulloblastoma tumour growth and angiogenesis.

**Methods:**

To this extent, we selected three SPARC full-length cDNA overexpressed clones (Daoy-SP). Consequences of SPARC overexpression were studied in terms of cell growth, angiogenesis using co-culture assay *in vitro*, dorsal skin-fold chamber assay *in vivo*, PCR Array for human angiogenic genes, as well as western blotting for angiogenic molecules and tumour growth, in an orthotopic tumour model.

**Results:**

The SPARC protein and mRNA levels were increased by approximately three-fold in Daoy-SP cells compared with parental (Daoy-P) and vector (Daoy-EV) controls. Daoy-SP clones reduced tumour cell-induced angiogenesis *in vitro* and *in vivo*, and formed small tumours with fewer blood vessels when compared with controls. Matrix metalloprotease-9 (MMP-9) and vascular endothelial growth factor (VEGF) expression were decreased in Daoy-SP clones. Further, inhibition of MMP-9 expression caused SPARC-mediated inhibition of angiogenesis and tumour growth as MMP-9 rescued SPARC-mediated anti-angiogenic effect *in vitro* and tumour growth inhibition *in vivo*.

**Conclusion:**

Overexpression of SPARC decreases angiogenesis, which leads to decreased tumour growth. Further, the role of MMP-9 could be attributed to the anti-angiogenic effect of SPARC.

Medulloblastoma (MB) is a malignant embryonal tumour that is predominantly diagnosed in children and continues to pose a difficult clinical management problem. Medulloblastomas occur principally in the midline cerebellar region but are prone to invade the meninges and cerebrospinal fluid spaces. With aggressive surgery, craniospinal radiotherapy and chemotherapy, only slightly >50% of children diagnosed with medulloblastoma are disease free after 5 years (Eberhart *et al*, 2002; Guerreiro *et al*, 2008; Packer and Vezina, 2008). Furthermore, current treatment for medulloblastoma carries a high risk of long-term morbidity, with the majority of patients having some degree of hormonal, auditory and neurocognitive impairment (Ris *et al*, 2001). Even with conventional chemotherapy and radiotherapy, only a subset of patients are cured; thus, a great need for new therapeutic approaches exists (Packer, 1999; Zeltzer *et al*, 1999). Similar to many other central nervous tumours, some PNET/MB tumours show marked neovascularisation (Assimakopoulou *et al*, 1997). Medulloblastomas produce a wide range of angiogenic factors that are, either individually or together, likely to have a direct role in tumour growth, tumour progression and angiogenesis (Pavlakovic *et al*, 2001a). Previous reports indicate that assessment of angiogenesis by microvessel density and quantification is significantly higher in medulloblastomas associated with poor prognosis (Ozer *et al*, 2004).

Secreted protein acidic and rich in cysteine (SPARC/osteonectin/BM-40) is a secreted macromolecule that interacts with cell-surface receptors, the extracellular matrix (ECM) and/or growth factors and proteases (Bornstein and Sage, 2002). The role of SPARC in tumourigenesis is complex and seems to be cell-type specific owing to its diverse functions in a given microenvironment. Solid tumours in SPARC-null mice grew significantly larger than those in wild-type animals (Brekken *et al*, 2003). The SPARC functions as a tumour suppressor in neuroblastoma, breast, pancreatic, lung and ovarian cancers (Framson and Sage, 2004). In addition, SPARC modulates angiogenesis and regulates the production, assembly and organisation of the ECM (Sage *et al*, 1984; Bradshaw and Sage, 2001). By directly binding to vascular endothelial growth factor (VEGF), SPARC inhibits microvascular endothelial cell proliferation stimulated by VEGF_165_, indicating that it has anti-angiogenic activity (Yan and Sage, 1999; Chlenski *et al*, 2006; Chandrasekaran *et al*, 2007). The SPARC binds to VEGF, thus inhibiting VEGF interaction with endothelial cell surface, VEGFR1 autophosphorylation, ERK1/2 activation and VEGF-induced DNA synthesis (Kupprion *et al*, 1998). The SPARC also binds platelet-derived growth factor-AB (PDGF-AB) and PDGF-BB on endothelial cells and inhibits the interaction of these growth factors with their tyrosine kinase receptors (Raines *et al*, 1992). In addition, SPARC influences the levels and activity of several angiogenic molecules (e.g., PDGF, fibroblast growth factor-2 (FGF-2), VEGF and insulin-like growth factor) (Francki *et al*, 1999, 2003; Brekken and Sage, 2001). Further, SPARC can inhibit angiogenesis indirectly by regulating the expression of other angiogenesis-related genes, such as matrix metalloproteases (MMPs) (Shankavaram *et al*, 1997) and transforming growth factor-*β* 1 (Francki *et al*, 1999). However, its role in tumour-induced angiogenesis seems to be tumour specific. Recent studies demonstrate that overexpression of SPARC led to a significant decline in microvessel density, resulting in delayed tumour formation and reduction in tumour size in hepatocellular carcinoma xenografts (Lau *et al*, 2006). The SPARC blocks angiogenesis *in vitro* and *in vivo* in neuroblastoma and is one of the key contributors to the anti-angiogenesis activity of the Schwann cell-conditioned medium (Chlenski *et al*, 2002). However, other studies point to the pro-angiogenic activity of SPARC. For example, SPARC was found high levels in breast cancer, colon cancer (Bellahcene and Castronovo, 1995; Porte *et al*, 1995), metastatic melanoma (Ledda *et al*, 1997) and invasive meningioma (Rempel *et al*, 1999).

In this study, we examined the effects of SPARC on medulloblastoma tumours *in vitro* and *in vivo* and whether inhibition of angiogenesis is implicated in the anti-tumour effect of SPARC. To elucidate the role of SPARC, we enhanced SPARC expression in medulloblastoma cells using stable transfection and expression constructs with SPARC full-length cDNA driven by a CMV promoter. We developed three human medulloblastoma cell lines designated as Daoy-SP1/2/3, which stably express human SPARC cDNA. The SPARC expression reduced xenograft growth with reduced vascularity in an orthotopic medulloblastoma model. We also demonstrated that SPARC expression inhibits VEGF-mediated angiogenesis by altering MMP-9 expression, thereby leading to reduced angiogenesis.

## Materials and methods

### Antibodies and reagents

Antibodies against SPARC, VEGF, epidermal growth factor receptor, fibroblast growth factor receptor (FGFR), PDGFR, VEGFR2, CD31, MMP-9 and major histocompatibility complex (MHC) class-I (Santa Cruz Biotechnology, Santa Cruz, CA, USA); Von-Willebrand factor (Factor-VIII) (DAKO Corp., Carpinteria, CA, USA); and MHC class-I antibody for immunohistochemistry (Serotec, Inc., Raleigh, NC, USA) were used. The RT^2^ PCR Array for angiogenesis (SA Biosciences, Frederick, MD, USA) was also used in this study. All other reagents were of analytical grade or better.

### Daoy cell culture

Daoy cells were obtained from ATCC (Manassas, VA, USA) and cultured in Advanced-MEM supplemented with 5% foetal bovine serum, 2 mM l^–1^ L-glutamine, 100 units ml^–1^ of each penicillin and 100 *μ*g ml^–1^ streptomycin. Cells were maintained in a humidified atmosphere containing 5% CO_2_ at 37°C.

### Construction of pcDNA3.1-SPARC and transfection of Daoy cells

An 1100-bp cDNA fragment of human SPARC was amplified by PCR using synthetic primers and sub-cloned into a pcDNA3.1 vector (Invitrogen, San Diego, CA, USA) in sense orientation. Daoy cells were transfected with full-length cDNA of SPARC containing vector or empty vector using FuGene HD (Roche, Indianapolis, IN, USA) as described earlier (Mohanam *et al*, 2001). Stable transfectants were selected with cloning cylinders after 3–4 weeks in medium containing G418. Wild-type Daoy cells are termed as Daoy parental (Daoy-P) and the stable cell lines overexpressing SPARC were designated as Daoy-SP, whereas Daoy-EV was the cell line transfected stably with the empty vector.

### Immunofluorescence microscopy

We used a previously described protocol with minor changes (Chetty *et al*, 2007). Briefly, cells were cultured on eight-well chamber slides and fixed with 4% (w/v) paraformaldehyde in phosphate-buffered saline (PBS), permeabilised with 0.1% (w/v) Triton X-100 in PBS and blocked with 1% (w/v) bovine serum albumin in PBS for 1 h at 4°C. Cells were incubated overnight either with anti-SPARC or with anti-Von-Willebrand factor antibody (1 : 100 dilution) at 4°C, followed by the corresponding Texas Red/fluorescent isothiocyanate (1 : 200 dilution)-conjugated secondary antibody for 1 h, and mounted with mounting medium containing 4,6-diamidino-2-phenylindole. The results were documented using a fluorescence microscope.

### Western blotting

Western blot analysis was performed as described previously (Bhoopathi *et al*, 2008). Briefly, 36 h after seeding, Daoy-P, Daoy-EV and Daoy-SP cells were collected and lysed in RIPA buffer. Equal amounts of proteins were resolved on SDS–polyacrylamide gel electrophoresis and transferred onto a PVDF membrane. The blot was blocked with 5% non-fat dry milk and probed overnight with primary antibodies (1 : 1000 dilution), followed by horseradish peroxidase-conjugated secondary antibodies (1 : 2500 dilution). An ECL system was used to detect chemiluminescent signals. All blots were re-probed with GAPDH antibody to confirm equal loading.

### siRNA design and transient transfection

The SPARC small interfering RNA (siRNA) sequences were designed with the help of a siRNA designer programme (Imgenex, Sorrento Valley, CA, USA). The siRNA was complementary to an exonic sequence of the target mRNA and compatible with the pcDNA3.1 vector (Invitrogen, San Diego, CA, USA). The following siRNA sequence 5′-TCGAGGGTGTGCAGCAATGACAACAAGAGTCGTCGTTGTTGTCATTGCTGCACACCG-3′ was used to construct SPARC siRNA. A control vector containing siRNA with a scrambled sequence was constructed and designated as pSV. We used the following scrambled sequence: 5′-CACGGAGGTTGCAAAGAATAATCGATTATTCTTTGCAACCTCCGTGC-3′. FuGene HD transfection reagent (Roche; 1 *μ*g plasmid: 3 *μ*l of FuGene HD) was used for transfections as per the manufacturer's instructions. After transfection, the cells were cultured in Advanced-MEM with 5% foetal bovine serum for 24 h, rinsed once with PBS and cultured for an additional 16 h in serum-free Dulbecco's modified Eagle's medium/F12 50/50. Conditioned medium and cell lysates were collected, and MMP-9 and SPARC levels were determined by gelatin zymography and western blot analysis, respectively.

### Reverse transcriptase PCR

Daoy-P, Daoy-EV and Daoy-SP cells were grown for 36 h. Total RNA was extracted from these cells and cDNA was synthesised using poly-dT primers as described earlier (Chetty *et al*, 2008). PCR was performed using the following primers: SPARC, 5′-GGAAGAAACTGTGGCAGAGG-3′ (sense) and 5′-ATTGCTGCACACCTTCTCAA-3′ (antisense); GAPDH, 5′-TGAAGGTCGGAGTCAACGGATTTGGT-3′ (sense) and 5′-CATGTGGGCCATGAGGTCCACCAC-3′ (antisense). Quantification of SPARC mRNA levels was carried out by densitometry.

### Cell proliferation assays

Cell proliferation was determined using a 3-(4,5-dimethylthiazol-2-yl)-2,5-diphenyl-2*H*-tetrazolium bromide (MTT) assay (R&D Systems, Minneapolis, MN, USA) as previously described (Rao *et al*, 2007). Briefly, Daoy-P, Daoy-EV and Daoy-SP (SP1, SP2 and SP3) cells (5000 cells per well) were seeded in 96-well cell culture plates and incubated up to 60 h. After 0–60 h, MTT reagent was added, incubated for 4 h at 37°C and the absorbance of formazan was measured with a microplate reader at *A*_550_.

### *In vitro* angiogenesis assay

Tumour cell-induced microtubule network formation was determined as described previously (Gondi *et al*, 2004). Daoy-P, Daoy-EV and Daoy-SP cells (2 × 10^4^ per well), either with SPARC siRNA, anti-SPARC antibody treatment or alone, were seeded in eight-well chamber slides and allowed to grow for 24 h. Thereafter, the medium was removed and HMEC cells (4 × 10^4^ cells per well) were added. After 36 h of co-culture, cells were stained with factor-VIII antigen. The formation of tubular capillary-like structures, which is an indicator of angiogenesis, was assessed by confocal microscopy. Image Pro Discovery software (Media Cybernetics, Inc., Bethesda, MD, USA) was used to quantify the tube length.

### Dorsal skin-fold chamber model

*In vivo* angiogenesis assay was performed as described previously (Lakka *et al*, 2005). Briefly, Daoy-P, Daoy-EV and Daoy-SP cells (1 × 10^6^ cells in 100 *μ*l) were injected into diffusion chambers (Fisher, Pittsburg, PA, USA) and sealed with bone wax. Athymic nude female mice (4–6 weeks old; five per group) were anaesthetised with ketamine:xylazine (100 : 10 mg kg^–1^). A dorsal air sac was created by subcutaneously injecting 5 ml of air, and a superficial incision was made at the edge of the air sac through which the chambers were placed underneath the skin. After 10 days, the animals were killed and the skin area covering the chambers was removed and photographed. The number of tumour cell-induced blood vessels was counted in five different fields as described previously (Lakka *et al*, 2005).

### RT^2^ profiler PCR array

Total RNA was isolated from Daoy-P, Daoy-EV and Daoy-SP cells and cDNA was synthesised as described above. The human angiogenesis signalling pathway RT^2^ Profiler PCR Array was used to profile the expression of 84 genes related to the angiogenesis signalling pathway, according to manufacturer's instructions. The fold change of mRNA expression was calculated on the basis of the cycle threshold (*C*_t_) values obtained from the real-time PCR experiment. The scatter plot of test *vs* control samples indicated the validity of the experiment.

### Intracranial tumour model and immunohistochemistry

All animal experiments were carried out after obtaining approval from the Institutional Animal Care and Use Committee on a project-specific basis in accordance with the Public Health Service Policy on Humane Care and Use of Laboratory Animals (PHS Policy), and meet the standards required by the UKCCCR guidelines (Workman *et al*, 1988). Animals were housed in pathogen-free conditions in a light/dark cycle of 12/12 h and fed with rodent chow and water *ad libitum*. Daoy cells with or without treatments (1 × 10^5^ cells, six animals per group, Daoy-P, Daoy-EV, Daoy-SP2, Daoy-EV+pcMMP9 and Daoy-SP2+pcMMP-9 cells) were stereotactically implanted as described previously (Bhoopathi *et al*, 2008). Animals were monitored for 180 days, which was the designated termination point of the experiment. Animals who lost >20% of body weight or had trouble ambulating, feeding or grooming were killed. For histological analysis, brains were snap frozen and maintained at −70°C until sectioning. Tumour volume was assessed as described previously (Ding *et al*, 2005). Briefly, all brains were serially sectioned, and 8-*μ*m sections were incubated with mAb anti-human-specific MHC class-I IgG (10 *μ*g ml^–1^, 2 h, at 4°C; mAb W6/32, Serotec, Inc.), followed by a multi-link secondary antibody conjugated to biotin and horseradish peroxidase, followed by incubation with streptavidin, and then 3,3′-diaminobenzidine substrate (Sigma, St Louis, MO, USA). Digital images were imported into Adobe Photoshop (San Jose, CA, USA), stained areas in each section were quantified as a pixel number and pixel numbers for all sections from each brain were summed to obtain a total pixel number. Excised brains were fixed in 10% buffered formalin and embedded in paraffin. Tissue sections (5 *μ*m thick) were obtained from the paraffin blocks and were stained with haematoxylin and eosin using standard histological techniques. For immunohistochemical analysis, sections were incubated with mAb (1 h, RT), followed by the appropriate secondary antibody. For horseradish peroxidase-conjugated secondary antibody incubation, we used 3,3′-diaminobenzidine solution as chromogen. Nucleus was counterstained with either haematoxylin or 4,6-diamidino-2-phenylindole. Negative control slides were obtained by nonspecific IgG. Sections were mounted with mounting solution and analysed using an inverted microscope.

### Statistical analysis

All data are expressed as mean±s.e. Statistical analysis was performed using Student's *t*-test or a one-way analysis of variance. A *P*-value of <0.05 was considered statistically significant. All experiments were performed in triplicate with consistent results.

## Results

### Overexpression of SPARC in Daoy cells

**Figure 1:**
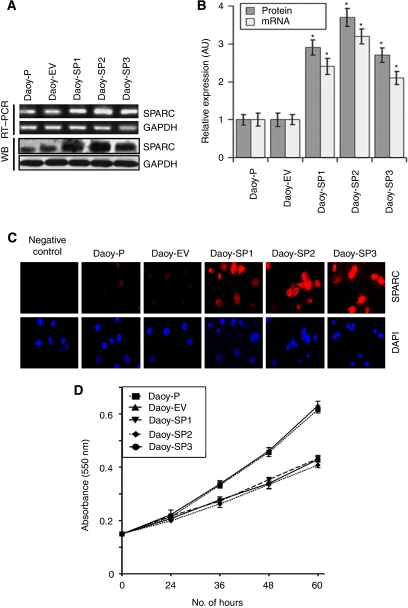
Overexpression of secreted protein acidic and rich in cysteine (SPARC) in Daoy medulloblastoma cells inhibits Daoy cell proliferation. Daoy cells were stably transfected with plasmid containing full-length SPARC cDNA and empty vector. (**A**) Total RNA was extracted using Trizol reagent, and reverse transcriptase PCR was performed for assessment of SPARC mRNA transcript level. GAPDH served as a control for RNA quality. SPARC protein levels were determined in total cell lysates by western blot analysis using SPARC-specific antibody. GAPDH was used to confirm equal loading of cell lysates. (**B**) Protein and mRNA transcripts level were quantified by densitometric analysis as shown in the corresponding bar graph. *Columns*, mean of triplicate experiments; *bars*, s.e.; ^*^P<0.01, significant difference from Daoy-P control cells. (**C**) Immunocytochemical analysis for SPARC in different SPARC-overexpressed clones. Mouse IgG was used as negative control. (**D**) Daoy-P, Daoy-EV and Daoy-SP cells (5000 cells) were plated in 96 well plates, incubated for 0–60 h and 20 *μ*l of 0.5 mg ml^–1^ 3-(4,5-dimethylthiazol-2-yl)-2,5-diphenyl-2*H*-tetrazolium bromide (MTT) in phosphate-buffered saline (PBS) were added to cells. The cells were incubated for another 4 h. Next, the medium was removed from each well and dimethyl sulphoxide (DMSO) (100 *μ*l) was added to each well to dissolve the formazan crystals. Absorbance values at 550 nm were measured with a microplate reader and the results were presented with the comparison of cells treated with vehicle. *Points*, mean of triplicate experiments; *bars*, s.e.

The SPARC, a prototype of the matricellular protein family, was shown to have an important role in various aspects of tumourigenesis, including tumour invasion, angiogenesis and tumour growth. To experiment with a genetic approach to induce SPARC expression and observe its effects on medulloblastoma tumour growth *in vitro* and *in vivo*, we cloned a human SPARC cDNA in a pcDNA3.1 mammalian expression vector and transfected it into Daoy parental (Daoy-P) cells. The stable cell lines overexpressing SPARC were designated as Daoy-SP, whereas the stable cell line expressing the empty vector (pcDNA3.1) was designated as Daoy-EV. We randomly tested clones for mRNA expression of SPARC transcript (data not shown) and selected three SPARC-overexpressed stable clones. Figure 1A indicates that SPARC transcript levels were increased in the three clones tested when compared with parental and vector controls. There was about a three-fold increase in mRNA transcript levels in Daoy-SP clones (Daoy-SP1, Daoy-SP2 and Daoy-SP3; *P*<0.01 *vs* controls; Figure 1B). To confirm that this upregulation of SPARC mRNA translated into increased levels of SPARC protein, we next performed western blot and immunocytochemical analyses for SPARC expression in these three Daoy-SP clones. We found a three- to four-fold increase in SPARC expression in Daoy-SP clones compared with parental and empty vector controls (*P*<0.01; Figure 1B). As assessed by immunofluorescence microscopy, the distribution of SPARC indicated intense staining in all three Daoy-SP clones, compared with Daoy-P and Daoy-EV controls (Figure 1C).

### Overexpression of SPARC decreases Daoy cell proliferation

To determine whether SPARC overexpression affected the growth of Daoy cells, the growth rates of SPARC-overexpressed cells were compared with those of parental and empty vector controls. A very minimal decrease in proliferation was observed at 24 h (5–8%). At 48 h, there was an ∼15% decrease in proliferation in all three SPARC-overexpressed clones, compared with Daoy-P and Daoy-EV cells. Finally, at 60 h, there was a 24, 30 and 25% inhibition of Daoy-SP1, Daoy-SP2 and Daoy-SP3 cells, respectively, compared with Daoy-P and Daoy-EV cells (Figure 1D).

### SPARC decreases tumour-induced angiogenesis *in vitro* and *in vivo*

**Figure 2:**
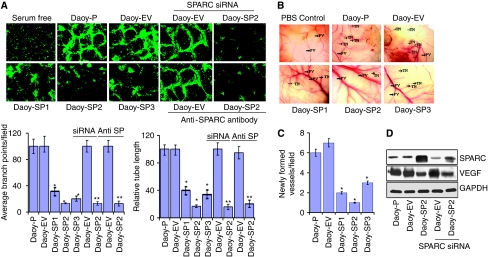
Overexpression of secreted protein acidic and rich in cysteine (SPARC) in Daoy cells inhibits tumour-induced angiogenesis *in vitro* and *in vivo*. (**A**) *In vitro* angiogenesis: Daoy-P, Daoy-EV and Daoy-SP cells (2 × 10^4^ per well), either with SPARC siRNA treatment or with anti-SPARC antibody treatment, were seeded in eight-well chamber slides. After 24 h, the medium was removed and 4 × 10^4^ HMEC cells were added. The cells were allowed to co-culture for 36 h the cells were fixed and performed immunofluorescence for factor-VIII as described in the ‘Materials methods’ section and observed for angiogenic response. Relative branch points and relative tube length were quantified as described in the ‘Materials and methods’ section. *Columns*, mean of triplicate experiments; *bars*, s.e.; ^*^P<0.01, significant difference from Daoy-P cells; ^**^P<0.01, significant difference from Daoy-EV cells treated with SPARC siRNA or anti-SPARC antibody (siRNA=SPARC siRNA; anti-SP=anti-SPARC antibody). (**B**) *In vivo* angiogenesis: Daoy-P, Daoy-EV and Daoy-SP cells (1 × 10^6^) were implanted into diffusion chambers and were surgically placed underneath the dorsal skin of athymic nude mice as described in the ‘Materials and Methods’ section. PV, pre-existing vasculature; TN, tumour-induced vasculature. (**C**) Newly formed vessels were quantified and represented as per field. *Columns*, mean of triplicate experiments; *bars*, s.e.; ^*^P<0.01, significant difference from Daoy-EV cells. (**D**) Western blot analysis showing SPARC and vascular endothelial growth factor (VEGF) levels with the treatment of siRNA against SPARC. GAPDH served as loading control.

Previous studies indicate that purified SPARC blocked endothelial cell migration in a dose-dependent manner in PNET tumours (Chlenski *et al*, 2002). To confirm this effect in SPARC-overexpressed Daoy cells, cells were grown for 24 h and then co-cultured with endothelial cells in an *in vitro* angiogenic assay as described in the ‘Materials and methods’ section; cell number was corrected for 5–8% inhibition at the 24 h time point in cell growth in Daoy-SP clones as compared with controls. Daoy-P and Daoy-EV cells cultured with endothelial cells elicited a strong angiogenic response and induced HMECs to differentiate into capillary-like structures within 36 h. In contrast, microvessel morphogenesis was impeded in the co-cultures of HMECs and Daoy-SP clones. Quantification indicated a 75–80% decrease in the formation of branch points and a 60–75% decrease in vessel length in HMEC cells cultured with Daoy-SP clones, compared with HMEC cells cultured with Daoy-P and Daoy-EV (Figure 2A).

We also examined whether Daoy-SP clones could inhibit tumour angiogenesis *in vivo* as assessed by the dorsal window model. Implantation of a chamber containing Daoy-P and Daoy-EV cells in the dorsal skin-fold chamber resulted in the development of tumour-induced microvessels (TN) with curved thin structures and many tiny bleeding spots. In contrast, implantation of Daoy-SP cells (cell number corrected for growth inhibition) had a 50–75% decrease in tumour-induced microvessels, compared with Daoy-P and Daoy-EV cells (Figures 2B and C).

To further test the effect of increased expression of SPARC on angiogenesis *in vitro*, we inhibited SPARC expression using siRNA or antibody against SPARC in Daoy-SP cells and tested their ability to induce endothelial cell network formation. Figure 2D indicates that SPARC expression in SPARC siRNA-transfected Daoy-SP cells was decreased compared with that in Daoy-SP cells and was comparable with that of Daoy-P parental controls. However, as shown in Figure 2A, siRNA against SPARC or anti-SPARC antibody could not restore Daoy-SP tumour cell-induced angiogenesis. Taken together, these data suggest that the decreased angiogenesis in SPARC-overexpressed cells could be because of SPARC-induced altered cellular composition rather than the effect of SPARC expression itself.

### Overexpression of SPARC modulates angiogenic factors

**Figure 3:**
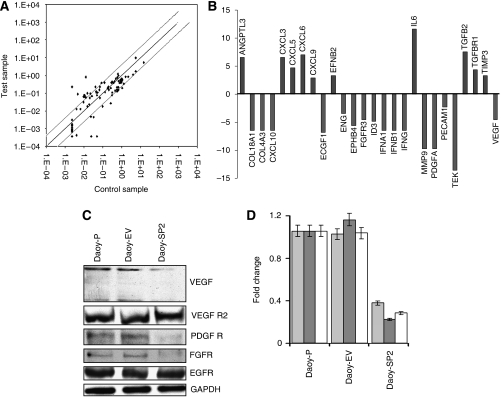
RT^2^ profiler PCR array for angiogenic factors in secreted protein acidic and rich in cysteine (SPARC)-overexpressed Daoy cells. We used the human angiogenesis signalling pathway RT^2^ Profiler PCR Array to profile the expression of 84 genes related to the angiogenesis signalling pathway. (**A**) The scatter plot of the test *vs* control samples indicates the validity of the experiment. (**B**) Graph showing decreased expression of several angiogenic factors and increased expression of anti-angiogenic factors. (**C**) Daoy-P, Daoy-EV and Daoy-SP2 cell lysates were used to perform western blot analysis for the angiogenic growth factors, i.e. vascular endothelial growth factor (VEGF), VEGFR2, platelet-derived growth factor receptor (PDGFR), fibroblast growth factor receptor (FGFR) and epidermal growth factor receptor (EGFR). GAPDH served as a loading control. (**D**) Protein levels were quantified by densitometry as shown in the corresponding bar graph. All experiments were performed in triplicate with consistent results. *Columns*, mean of triplicate experiments; *bars*, s.e.; ^*^P<0.01, significant difference from Daoy-P cells.

To better determine the mechanisms underlying SPARC-mediated inhibition of angiogenesis, we used the human angiogenesis signalling pathway RT^2^ Profiler PCR Array to profile the expression of 84 genes related to the angiogenesis signalling pathway. Total RNA from Daoy-P, Daoy-EV and Daoy-SP2 cells (Daoy-SP2 cells exhibited higher anti-angiogenic effect than Daoy-SP1 and Daoy-SP3) was used to synthesise cDNA. Real-time PCR was performed as per the manufacturer's instructions and fold change of mRNA expression was calculated on the basis of *C*_t_ values. The scatter plot of the test (Daoy-SP2) *vs* control (Daoy-EV) samples indicates the validity of the experiment (Figure 3A). It is evident from the results that SPARC overexpression led to decreased expression of pro-angiogenic factors (e.g., VEGF, FGFR, ECGF and MMP-9), as well as increased expression of anti-angiogenic factors (e.g., TIMP-3 and transforming growth factor-*β*; Figure 3B). To confirm the PCR array results, protein levels of pro-angiogenic molecules in cell lysates from Daoy-P, Daoy-EV and Daoy-SP2 cells were assessed using western blotting (Figure 3C). When adjusted for parental controls, densitometry analysis revealed a 65, 78 and 70% decrease in VEGF, PDGFR and FGFR, respectively, in Daoy-SP2 cells compared with controls (Figure 3D). The VEGFR2 and epidermal growth factor receptor expression remained unchanged (Figures 3C and D).

### Overexpression of SPARC inhibits MMP-9-mediated angiogenesis activity and VEGF levels

**Figure 4:**
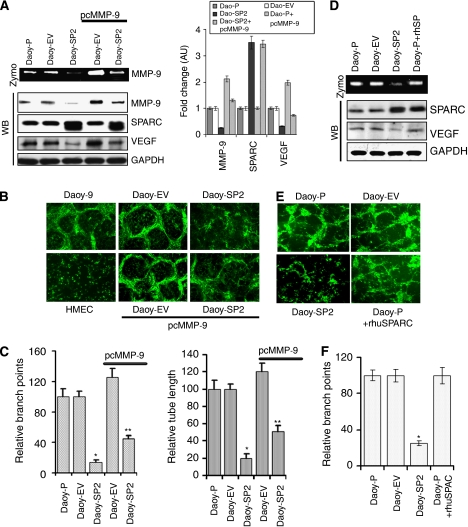
Overexpression of secreted protein acidic and rich in cysteine (SPARC) inhibits matrix metalloprotease-9 (MMP-9) activity and reduces vascular endothelial growth factor (VEGF) protein levels. (**A**) Daoy-P, Daoy-EV and Daoy-SP2 cells were transiently transfected with a vector encoding full-length MMP-9 cDNA (pcMMP-9). Gelatin zymography was carried out for MMP-9 activity. Western blot analysis was performed for MMP-9, SPARC and VEGF protein levels. The blots were stripped and reprobed with GAPDH antibody to detect total amounts of the respective proteins. (**B**) Daoy-P, Daoy-EV and Daoy-SP2 cells (2 × 10^4^ per well) were seeded in eight-well chamber slides transfected with pcMMP-9 and allowed to grow for 24 h. Then, the medium was removed and 4 × 10^4^ HMEC cells were added. The cells were allowed to co-culture for 36 h the cell are fixed and performed immunofluorescence microscopy for factor-VIII as described in the ‘Materials and methods’ section and observed for angiogenic response. (**C**) Angiogenic result was quantified by counting the relative branch points and tube length in five different fields from three independent experiments. *Columns*, mean of triplicate experiments; *bars*, s.e.; ^*^P<0.01, significant difference from Daoy parental cells; ^**^P<0.01, significant difference from Daoy-SP cells without pcMMP-9 treatment. (**D**) Parental Daoy cells were exogenously treated with recombinant human SPARC (rhuSPARC) for 36 h. Gelatin zymography was carried out for MMP-9 activity. Western blot analysis was performed for SPARC and VEGF protein levels. The blots were stripped and reprobed with GAPDH antibody to detect total amounts of the respective proteins. (**E**) Daoy-P, Daoy-EV and Daoy-SP2 cells (2 × 10^4^ per well) were seeded in eight-well chamber slides and Daoy-P cells were treated with rhuSPARC and allowed to grow for 24 h and performed co-culture assay as described above. (**F**) Angiogenic result was quantified by counting the relative branch points from three independent experiments. *Columns*, mean of triplicate experiments; *bars*, s.e. ^*^P<0.01, significant difference from Daoy-P cells.

The SPARC and MMP-9 are known to interact to regulate many stages of tumour progression including ECM deposition, angiogenesis and metastasis (Arnold *et al*, 2008). In addition, as mentioned above, the RT^2^ Profiler PCR Array for angiogenesis and western blot analysis showed that SPARC overexpression led to decreased expression of MMP-9 and VEGF (Figure 3B). We therefore examined the possible role of MMP-9 in the anti-angiogenic effect of SPARC-overexpressed Daoy medulloblastoma cells. Our results show that MMP-9 activity and protein expression were decreased in Daoy-SP2 cells, compared with Daoy-P and Daoy-EV cells (Figure 4A). In addition, the expression of VEGF, which has an essential role in endothelial proliferation and angiogenesis, was also decreased in Daoy-SP2 cells compared with parental and vector controls. We next examined the ability of MMP-9 expression in Daoy-SP2 cells to induce angiogenesis *in vitro*. Figure 4B indicates that ectopic expression of MMP-9 using a vector expressing full-length MMP-9 cDNA in Daoy-SP2 cells induced MMP-9 expression, which is comparable to that of parental Daoy-P cells. Further, VEGF expression was also induced in these cells, indicating that VEGF expression is dependent on MMP-9 expression in SPARC-overexpressed cells (Figure 4A). To elucidate the role of MMP-9 in SPARC-mediated inhibition of tumour cell-induced angiogenesis, we performed real-time PCR array for angiogenesis and western blot analysis with MMP-9 overexpression in Daoy-SP cells (Daoy-P *vs* Daoy-SP2+pcMMP9). The results indicated that MMP-9 overexpression in Daoy-SP2 cells increased angiogenic factors and led to increased angiogenesis. When adjusted for parental controls, densitometry analysis indicated that VEGF, FGFR and PDGFR were increased by 60, 65 and 68%, respectively, in Daoy-SP2 cells treated with pcMMP-9 when compared with Daoy-SP2 cells. (Supplementary Figure 1).

To further characterise the role of MMP-9 in SPARC-mediated inhibition of angiogenesis, we performed an *in vitro* angiogenic assay with Daoy-SP2 cells transfected with MMP-9 cDNA. Figures 4B and C shows that ectopic expression of MMP-9 in Daoy-SP2 cells reversed the anti-angiogenic effects of these cells. We exogenously added human recombinant SPARC protein and determined weather Daoy tumour cells could induce tumour cell-induced angiogenesis. Our data indicate that addition of exogenous SPARC did not inhibit tumour cell-induced angiogenesis, unlike the forced expression of SPARC (Figure 4E). In addition, supplementing recombinant SPARC to Daoy-P cells, comparable with that of the SPARC levels in DAOY-SP2 cells, did not alter the angiogenic capability of these cells. Further addition of recombinant SPARC did not change the expression of MMP-9 and VEGF, as observed in the case of SPARC-overexpressed Dapy-SP2 cells (Figure 4D). These observations, taken together with the studies presented in Figure 2, confirm that SPARC expression-mediated anti-angiogenic effects are due to altered gene expression rather than due to the expression of SPARC itself.

### Overexpression of SPARC in medulloblastoma cells inhibits tumourigenicity in nude mice

**Figure 5:**
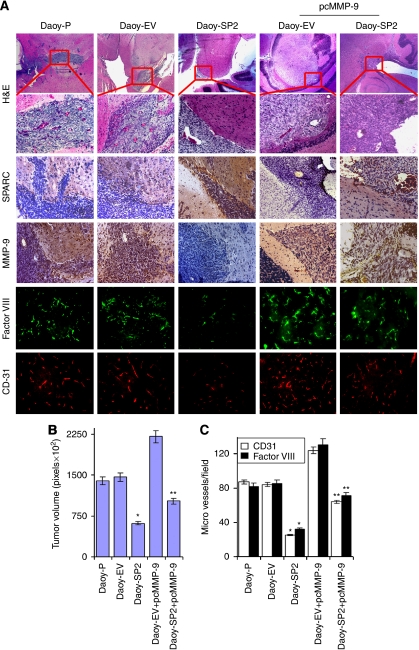
Secreted protein acidic and rich in cysteine (SPARC) inhibits medulloblastoma tumour growth *in vivo*. Medulloblastoma tumour sections from mice injected with Daoy-P, Daoy-EV, Daoy-SP2, Daoy-EV+pcMMP-9 and Daoy-SP2+pcMMP-9 cells were analysed as described in the ‘Materials and Methods’ section. (**A**) *Haematoxylin and eosin (H&E) staining for the tumours*: paraffin-embedded sections of tumours were used for immunohistochemical analysis for SPARC, MMP-9, CD-31 and factor-VIII was performed as described in the ‘Materials and Methods’ section. (**B**) Tumour volume was quantified and the results are presented in pixels. (**C**) CD31 (red) and factor-VIII (green) positive areas were counted and quantified as per microscopic field and results are represented. *Columns*, mean of triplicate experiments; *bars*, s.e.; ^*^P<0.01, significant difference from Daoy parental cells. ^**^P<0.05, significant difference from Daoy-SP2 cells. The colour reproduction of the figure is available on the html full text version of the paper.

To assess the therapeutic efficacy of SPARC expression, Daoy-P, Daoy-EV and Daoy-SP2 cells were injected intracranially into nude mice. Mice injected with Daoy-P and Daoy-EV cells developed tumours, became symptomatic within 6 weeks and were subsequently killed. In striking contrast, mice injected with Daoy-SP2 cells survived for 180 days, which was the designated end point of the experiment. At this point, the animals were killed and their brains were examined for tumour growth. Tumours volumes were evaluated by measuring the maximum cross-sectional areas stained for anti-human-specific MHC class-I IgG (western blot analysis of MHC class-I IgG in tumours is shown (Supplementary Figure 3) in digitalised sections of cerebellum/tumour. Haematoxylin and eosin staining indicates significant tumour growth in brains of mice implanted with Daoy-P and Daoy-EV cells (Figure 5A). A corresponding statistically significant decrease (∼65%) in mean tumour volume was found in animals implanted with Daoy-SP2 (*n*=6; mean tumour volume=54 000 pixels±7000) as compared with animals implanted with Daoy-EV (*n*=6; mean tumour volume=156 000 pixels±16 000; *P*<0.001).

To determine whether SPARC was expressed *in vivo*, brain sections were stained with a monoclonal antibody for human SPARC. Figure 5A indicates that tumour sections from Daoy-SP2 tumours showed more staining than did Daoy-P and Daoy-EV tumours. To assess whether MMP-9 inhibition mediated the anti-tumour effects of SPARC expression, we analysed angiogenesis in xenografts by histological analysis for CD-31, factor-VIII and MMP-9 expression (Figure 5A). Estimation of CD-31 and factor-VIII-positive areas in 20 randomly selected fields of tumours from Daoy-EV and Daoy-SP provided some measure of vascularity in the tumour sections (Figure 5C). Only a small fraction (15–20%) of CD-31- or factor-VIII-positive areas remained in Daoy-SP2 tumours in comparison with the controls. It was also evident that the overexpression of SPARC in tumours caused a decrease in MMP-9 expression in these tumours (Figure 5A).

We also analysed the effect of MMP-9 on the *in vivo* consequences of SPARC expression on medulloblastoma tumour formation in nude mice. Daoy-SP2 cells were transfected with MMP-9 cDNA and were injected into the brains of nude mice, and the formation of tumours was monitored. As shown in Figure 5A, ectopic expression of MMP-9 using a vector expressing full-length *MMP-9* gene in Daoy-SP2 cells induced MMP-9 expression comparable with that of parental Daoy-P cells. The MMP-9 induction reversed SPARC-mediated tumour growth inhibition by 50%, with a mean tumour volume of 30% of the empty vector control (Figure 5B). Accordingly, MMP-9, CD31 and factor-VIII expression were increased in the tumour sections in these mice compared with the tumour sections of mice with Daoy-SP2 cells (Figure 5C). These results are consistent with a role for MMP-9 in the SPARC-induced anti-angiogenic effect observed *in vitro*.

## Discussion

The SPARC is an ECM protein that influences the ‘soil’ in which tumours develop. The ECM is known to influence tumour growth directly (Netti *et al*, 2000; Hotary *et al*, 2003). The various activities associated with SPARC are thought to facilitate different steps in the formation of new vessels (Brekken and Sage, 2000). This study reports that SPARC expression results in a significant decrease in angiogenic capacities *in vitro* and *in vivo* and remarkably reduces *in vivo* tumourigenicity of medulloblastoma cells.

The contradictory reports regarding the role of SPARC in cell growth and tumour formation suggest that its effects are cell-type specific and may be dependent on concentration and ECM components. Therefore, the focus of this investigation was to determine the role of SPARC in medulloblastoma (i.e., the possibility of its use as a therapeutic target or agent). Using the Daoy cell line stably transfected with SPARC cDNA, we determined the contribution of SPARC in medulloblastoma tumour growth. SPARC protein and gene transcript levels were increased by about three-fold in Daoy-SP clones compared with Daoy-P and Daoy-EV stable clones, as determined by western blotting and reverse transcriptase PCR analyses. The SPARC expression inhibited tumour cell-induced tube formation by endothelial cells in an *in vitro* co-culture assay and abrogated induction of angiogenesis in a dorsal window air sac assay *in vivo.* To investigate the function of SPARC in the regulation of medulloblastoma tumour growth *in vivo,* Daoy-SP cells were compared for their ability to form tumours in an intracranial model. Overexpression of full-length SPARC significantly reduced tumour size in xenografts. Therefore, in medulloblastoma, SPARC expression is inversely correlated with malignant phenotype. Previous studies have indicated that SPARC contributes to the regulation of tumour formation, although its role seems to be cell-type specific. The SPARC exerts growth inhibitory activity on immortalised ovarian cancer cells *in vitro* and in mouse xenograft explants (Abeysinghe *et al*, 2003). Similarly, the tumour growth of glioma cells overexpressing SPARC was delayed *in vivo* (Schultz *et al*, 2002). An inhibitory effect of SPARC on proliferation and migration has been found in breast and ovarian carcinoma cells (Dhanesuan *et al*, 2002). Infection of MDA-231 breast carcinoma cells with osteonectin decreased the *in vitro* invasion of these cells through Matrigel (Koblinski *et al*, 2005). Overexpression of SPARC by ovarian carcinoma cells led to increased tumour cell apoptosis, and the levels of SPARC were inversely correlated with tumour progression *in vivo* (Yiu *et al*, 2001). The growth of Lewis lung carcinoma and B-cell lymphoma was enhanced in mice lacking endogenous SPARC (Brekken *et al*, 2003). Pancreatic tumour growth was enhanced in mice lacking endogenous SPARC (Puolakkainen *et al*, 2004).

Tumour growth has been shown to depend on angiogenesis. Our study demonstrates that the anti-tumour effect of SPARC expression *in vivo* is mediated, at least in part, by anti-vascular effects. The reduced tumour growth in tumours formed with Daoy cells expressing SPARC cDNA was associated with a decrease in angiogenesis. Medulloblastomas produce a wide range of angiogenic factors that are likely to have a direct role in tumour growth (Pavlakovic *et al*, 2001a). Besides its effect on proliferation, SPARC is thought to function in tissue remodelling and angiogenesis. However, the role of SPARC on tumour cell-induced angiogenesis has not been clearly established. Our results show that there was reduced capillary tube formation when endothelial cells were grown in the presence of Daoy-SP cells as compared with the parental and vector controls in an *in vitro* angiogenic assay. In addition to these *in vitro* anti-angiogenic activities, SPARC seems to be anti-angiogenic *in vivo*, as demonstrated by the suppression of neovascularisation in the dorsal window air sac assay. Analysis of tumours formed with SPARC-overexpressing clones showed that SPARC overexpression reduces vessel density as determined by CD31 or factor-VIII staining. These results confirm that SPARC is an inhibitor of tumour angiogenesis *in vivo*.

A variety of growth factors can stimulate angiogenesis. The most important angiogenesis stimulators are VEGF (Veikkola and Alitalo, 1999) and bFGF (Pavlakovic *et al*, 2001b). Tumour cells may overexpress one or more of these angiogenic factors, which in turn function synergistically to promote tumour growth. It has been reported that some of the primitive neuroectodermal tumours, which include medulloblastoma, show immunoreactivity for VEGF and bFGF (Brem *et al*, 1992; Pietsch *et al*, 1997). In another study, as determined by immunohistochemistry, VEGF was expressed in all tested medulloblastoma tumours, suggesting that inhibition of VEGF in medulloblastomas may result in the inhibition of angiogenesis, as well as in tumour growth and progression (Ozer *et al*, 2004). The relationship between SPARC in tumour cells and the expression of angiogenic molecules, as evaluated using a PCR array, indicated that several angiogenic molecules were reduced in Daoy-SP cells compared with controls. Similarly, results from the western blot analysis also showed the downregulation of some potent angiogenic molecules, including MMP-9, VEGF, PDGFR and FGF. These results suggest that SPARC expression alters the angiogenic balance in the tumour microenvironment by altering the expression of a complex array of inhibitors and stimuli. Growth factors, such as VEGF or PDGF, act directly on endothelial cells and/or activate inflammatory cells (monocytes and T lymphocytes), which in turn synthesise angiogenic factors. Previous studies demonstrated that SPARC regulates additional components and coordinates the activity of growth factors on endothelial cell proliferation and migration. The SPARC antagonised the migratory response of endothelial cells to bFGF without the binding of SPARC to bFGF or the blocking of the ligand–receptor interaction (Hasselaar and Sage, 1992). The SPARC modulates glioma growth by altering the tumour microenvironment and suppressing tumour vascularity through suppression of VEGF expression and secretion. (Yunker *et al*, 2008). In this context, the suppression of angiogenesis-mediated tumour growth by SPARC in medulloblastoma seems to be the consequence of its ability to inhibit the expression of angiogenic factors such as MMP-9, bFGF and VEGF in tumour tissues, which may in turn inhibit capillary infiltration into tumours. We show that MMP-9 expression in SPARC-overexpressed clones restored VEGF expression and SPARC-induced anti-angiogenic effect. A positive feedback regulation between MMP-9 and VEGF was reported in retinal pigment epithelial cells (Hollborn *et al*, 2007). Our PCR array analysis also indicates that MMP-9 induction induced HIF expression (Supplementary Figure 1), a transcriptional factor for VEGF (Hellwig-Burgel *et al*, 2005) that probably induced VEGF levels.

Our results indicate that SPARC expression inhibits tumour growth *in vivo*. We also show that this inhibition of tumour growth could be mediated in part by inhibition of MMP-9. Our data also indicate that MMP-9 and VEGF levels were maximally inhibited in Daoy-SP2 cells, which show an increased anti-angiogenic effect compared with other clones (Supplementary Figure 2). Our studies indicate MMP-9 induction in Daoy-EV cell-induced angiogenesis, as indicated by an increase in branch points and enhanced tumour growth by 50% compared with that of controls. However, MMP-9 expression in Daoy-SP2 cells reversed the SPARC-mediated inhibition of anti-angiogenic effect and tumour growth by only about 50% compared with that of tumour cell-induced angiogenesis and tumour growth *in vivo* induced by Daoy-SP2 cells, although western blot analysis for MMP-9 indicated that transfection with plasmid expressing MMP-9 cDNA in Daoy-SP2 cells restored the expression of MMP-9 to that of parental cells (Figure 4A). In addition, western blot analysis of VEGF expression indicated that VEGF levels in MMP-9-induced Daoy-EV cells are almost two-fold higher than that of parental control cells, which probably contributed to the enhanced tumour growth induced by these cells. However, VEGF expression in MMP-9-induced Daoy-SP cells was restored to only about 70%. This suggests that factors other than MMP-9 and VEGF are involved in SPARC-mediated inhibition of tumour growth. Previous studies also indicate that SPARC alters the expression of several genes involved in cell-cycle progression, signalling and migration (Golembieski and Rempel, 2002). Moreover, we also show that SPARC inhibition with SPARC siRNA or antibody *in vitro* did not reverse the effect of SPARC-mediated inhibition of angiogenesis. In addition, exogenous addition of recombinant SPARC did not alter angiogenesis or MMP-9 and VEGF levels (Figure 4D). These studies suggest that decreased angiogenesis in SPARC-overexpressed cells could be due to SPARC-induced altered cellular composition rather than the effect of SPARC expression itself.

Little progress has been made in the development of molecular targeted therapies for paediatric malignancies. This study provides an insight into the possible functional roles of SPARC in medulloblastoma tumour angiogenesis, and the data demonstrate that SPARC expression is inversely correlated with medulloblastoma tumour growth *in vivo*.
