# Supplementary figures and images for: The role of MMP-9 in the anti-angiogenic effect of secreted protein acidic and rich in cysteine
Source: Br J Cancer. 2010 Jan 19;102(3):530–40. doi: 10.1038/sj.bjc.6605538 (PMC2822952; doi:10.1038/sj.bjc.6605538)

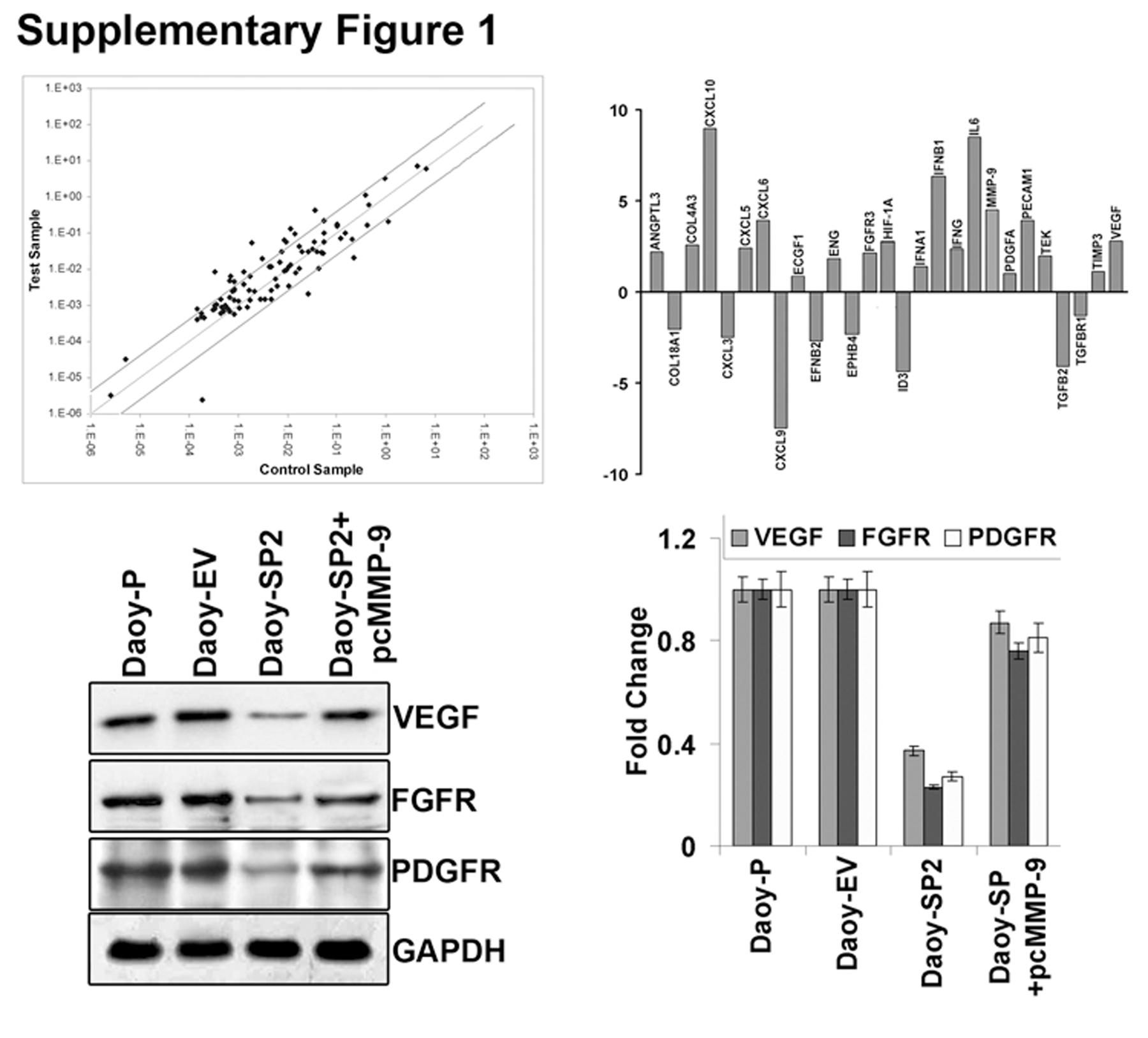

Supplement: Supplementary Figure 1 [file 6605538x1.tif]

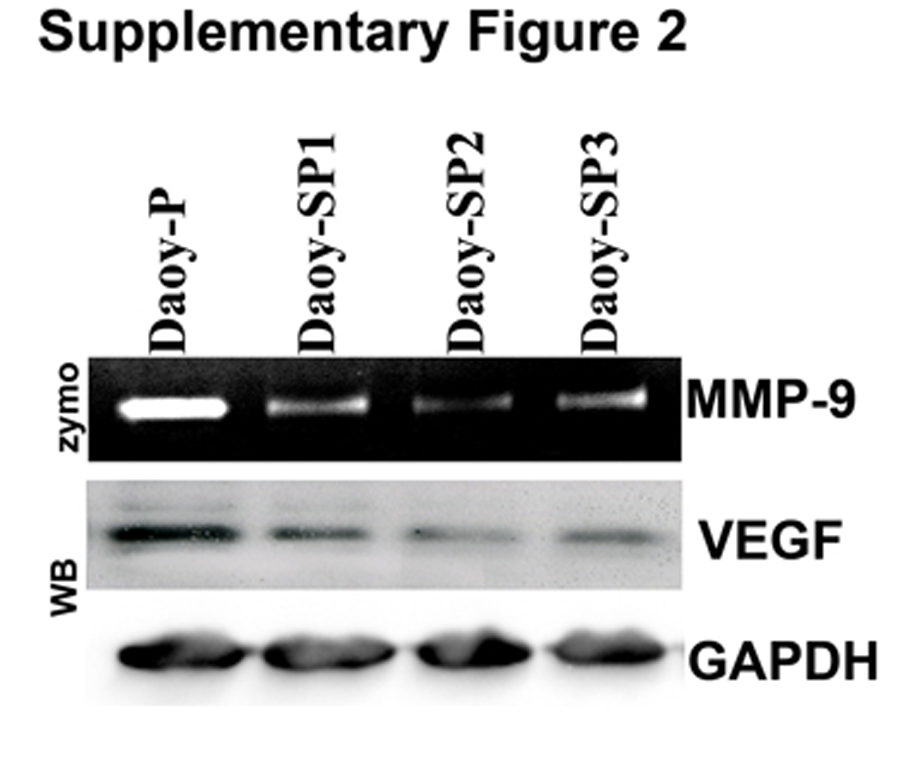

Supplement: Supplementary Figure 2 [file 6605538x2.tif]

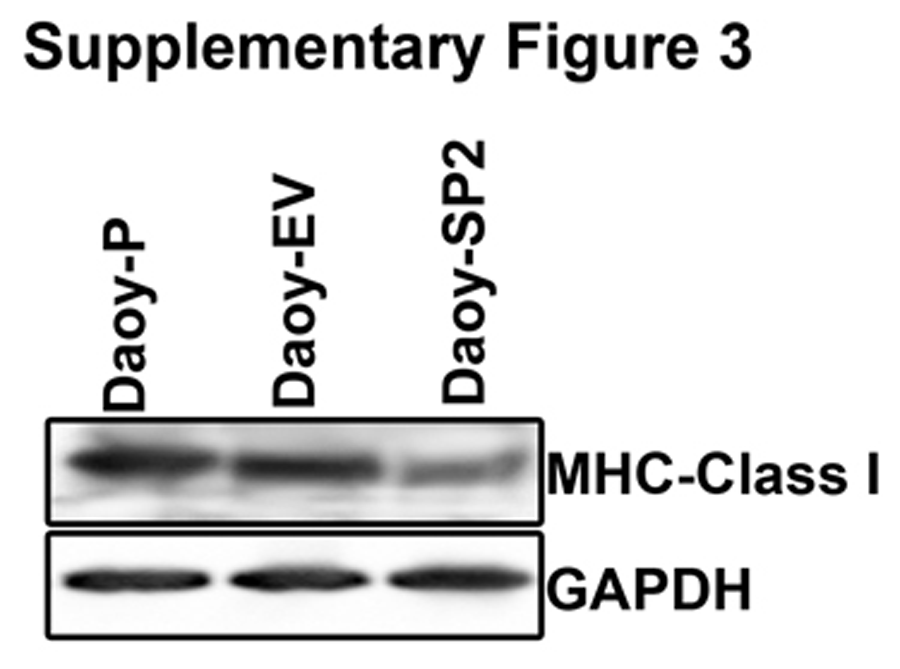

Supplement: Supplementary Figure 3 [file 6605538x3.tif]
